# Hand-Held Ultrasound Devices Compared with High-End Ultrasound Systems: A Systematic Review

**DOI:** 10.3390/diagnostics9020061

**Published:** 2019-06-15

**Authors:** Alexander Rykkje, Jonathan Frederik Carlsen, Michael Bachmann Nielsen

**Affiliations:** Department of Radiology, Copenhagen University Hospital, Rigshospitalet, 2100 Copenhagen, Denmark; jonathan.carlsen@gmail.com (J.F.C.); mbn@dadlnet.dk (M.B.N.)

**Keywords:** pocket devices, portable, hand-held, ultrasound, comparison, pleura, abdomen

## Abstract

The aim of this study was to review the scientific literature available on the comparison of hand-held ultrasound devices with high-end systems for abdominal and pleural applications. PubMed, Embase, Web of Science and Cochrane were searched following Preferred Reporting Items for Systematic Reviews and Meta-Analyses (PRISMA) guidelines. Original research describing hand-held ultrasound devices compared with high-end systems was included and assessed using Quality Assessment of Diagnostic Accuracy Studies (QUADAS)-2. The search was limited to articles published since 1 January 2012. A total of 2486 articles were found and screened by title and abstract. A total of 16 articles were chosen for final review. All of the included articles showed good overall agreement between hand-held and high-end ultrasound systems. Strong correlations were found when evaluating ascites, hydronephrosis, pleural cavities, in detection of abdominal aortic aneurysms and for use with obstetric and gynaecological patients. Other articles found good agreement for cholelithiasis and for determining the best site for paracentesis. QUADAS-2 analysis suggested few risks of bias and almost no concerns regarding applicability. For distinct clinical questions, hand-held devices may be a valuable supplement to physical examination. However, evidence is inadequate, and more research is needed on the abdominal and pleural use of hand-held ultrasound with more standardised comparisons, using only blinded reviewers.

## 1. Introduction

Ultrasound has provided valuable, non-invasive diagnostic images for decades. Technological advances in ultrasonography have led to gradually improved image quality on increasingly powerful machines. Despite their diagnostic usefulness, such high-end ultrasound systems are expensive, can be difficult to transport, and are often only available at highly specialised hospital wards [1]. In recent years smaller and increasingly portable medical ultrasound devices have been developed. Today, the smallest ultrasound devices can be carried in the pocket of a physician’s lab coat. Such hand-held devices are cheaper than high-end ultrasound systems and could potentially be more readily available, with each physician carrying his or her own device [2].

There are an increasing number of hand-held ultrasound devices on the market, each of them with different features. Some have the transducer and screen joined as one unit; others have a transducer connected to a tablet or smartphone, and some are wirelessly connected [3]. Hand-held devices have been tested for various clinical applications, such as in emergency care and as a ward-based supplement to the physical examination [4,5,6,7]. Other studies have compared results by novices with those of expert physicians [8,9]. It was not the focus of these studies to compare their results directly with high-end machines. The most thoroughly researched applications for hand-held ultrasound have been for use in echocardiography, but for many patients, ultrasound of the abdomen and the pleura can be the first diagnostic step to confirm or rule out initial diagnoses. The fast development of hand-held ultrasound devices could potentially make them a game-changer in the availability and cost of ultrasound examinations. It is, however, still to be decided if hand-held devices will change or speed up the clinical workflow in some of these cases.

The aim of this study is to provide a systematic review of the literature available on the comparison of hand-held devices with high-end ultrasound systems in the fields of abdominal and pleural applications.

## 2. Materials and Methods

We performed a systematic literature review following PRISMA guidelines (Preferred Reporting Items for Systematic Reviews and Meta-Analyses) [10]. The databases of PubMed, Embase, Web of Science and Cochrane Library were used for the literature search, which took place on 16 November 2018. The search string used was ("hand held" OR hand-held OR handheld OR portable OR pocket) AND (ultrasound OR ultrason* OR sonogra*) AND (system OR device) and was the same in all databases. The asterisk character (*) was used to match more than one word ending.

Only articles in English were considered and the search was further limited to articles published since 1 January 2012 (01.01.2012–16.11.2018). Duplicates were removed after which two authors (A.R. and J.F.C.) screened the search results by title and abstract. Only articles directly comparing hand-held devices with a high-end ultrasound system were included. Further, only articles concerning abdominal and/or pleural ultrasound were included. Articles concerning somewhat portable devices the size of a laptop computer were excluded from the review. All animal studies were excluded, so that only clinical studies conducted on humans were considered for this review. After the screening of abstracts, the eligible full text articles were read by the same two authors. Any discrepancies between the two reviewers were resolved by consensus. All reference lists of the included articles were searched manually for further eligible articles.

**Figure 1:**
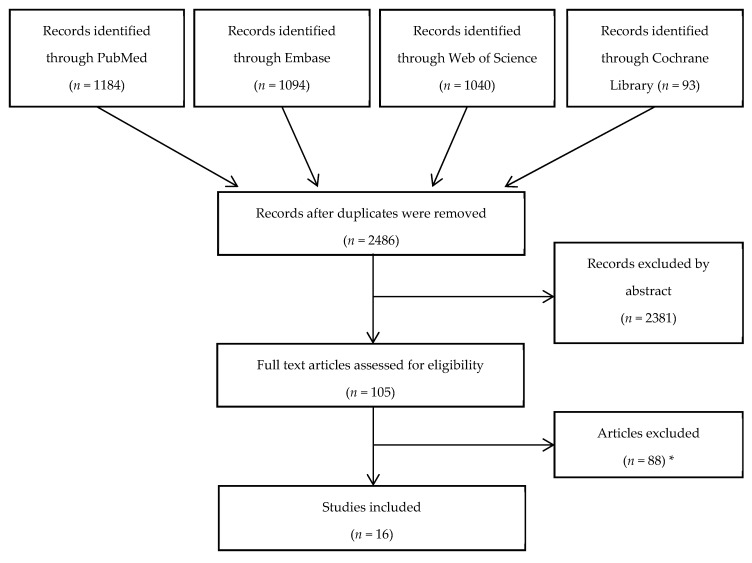
Flow chart following Preferred Reporting Items for Systematic Reviews and Meta-Analyses (PRISMA) guidelines. * Of the 88 articles excluded, 47 were conference abstracts or letters, 19 did not compare with a high-end ultrasound system, 9 did not use a true hand-held ultrasound device, 8 were not related to abdominal or pleural applications, three were not in English and two were conducted on animals.

The search yielded a total of 2486 articles after duplicates were removed (Figure 1). In total, 2381 articles were excluded by screening titles and abstracts. Of the 105 articles chosen for further full text evaluation, a total of 16 articles were included for final assessment in this review.

Authors, publication year, study design, aim, participant information, devices used (hand-held and high-end), main results and conclusions were registered in our study. To assess the applicability and validity of each study we used the QUADAS-2 tool (Quality Assessment of Diagnostic Accuracy Studies) [11].

## 3. Results

### 3.1. Study Overview

The included articles are presented in Table 1. Of the 16 included articles [12,13,14,15,16,17,18,19,20,21,22,23,24,25,26,27], four were related to the chest and lungs [12,13], five focused on abdominal ultrasound [14,15,16,17,18], two were related to urology [19,20], four to gynaecology and obstetrics [21,22,23,24] and three to vascular topics [25,26,27].

### 3.2. Devices

Fifteen of the 16 included articles used the hand-held device Vscan (GE Medical) [12,13,14,15,17,18,19,20,21,22,23,24,25,26,27] and one article used the device Acuson P10 (Siemens) [16]. The high-end ultrasound systems used in the included studies comprised of at least 24 different systems.

### 3.3. Operator Numbers, Experience and Study Workflow

The level of experience of operators using hand-held devices varied a lot between articles, ranging from medical students to expert physicians. Six articles had experienced physicians or sonographers operating all ultrasound devices [17,19,20,22,25,27]. Five studies provided a training period of a few days or weeks before comparison with a high-end system for either nurses, family physicians, medical residents or medical students [12,13,15,18,26]. One article compared the hand-held examinations of both experienced and non-experienced operators with those made on high-end ultrasound systems [14]. 

In twelve of the included articles, experienced physicians or sonographers performed the reference evaluation on high-end ultrasound systems [12,13,14,15,17,18,19,20,22,25,26,27]. One study divided operators using both systems into groups based on experience [24]. Finally, three studies had the same experienced investigators use both the hand-held and high-end ultrasound systems, documenting their findings immediately after the examinations [16,21,23]. 

The number of different operators on both hand-held and high-end systems also varied a great deal between the studies. One study included 11 sonographers [19] whilst another study had one operator do all examinations [23]. Some articles made use of only one operator in each group [14,17,20,22,27], some had several [12,13,15,16,21,26], and the rest did not specify how many operators there were for each ultrasound system [18,24,25].

In nine of the included articles the examination using the hand-held ultrasound device was performed first and followed by reference imaging with a high-end system [12,16,18,19,20,21,23,24,25]. Five studies began with the high-end ultrasound system followed by the hand-held device examinations [14,15,17,22,27]. In two articles it was unclear which type of device was used first [13,26]. In twelve of the included articles, both the hand-held- and high-end ultrasound examinations were performed on the same day. For the remaining six studies it was unclear how long a time interval there was between the scans [13,15,16,17,26,27].

### 3.4. Pleura (Two Articles)

Both articles investigating the pleural applications of hand-held ultrasound showed good overall agreement when comparing with high-end ultrasound systems. Patients were either hospitalised [13] or seen in an outpatient clinic [12]. 

Dalen et al. found a high sensitivity, specificity and positive and negative predictive values when evaluating pleural effusion with a hand-held device, and moderate sensitivity but high positive and negative predictive values when assessing the impact of heart failure on the inferior vena cava [12]. Graven et al. had cardiac nurses successfully assess and obtain reliable measurements of the pleural and pericardial cavities in all participating patients [13]

### 3.5. Abdomen (Five Articles)

All five articles related to the abdominal region found that hand-held ultrasound devices were in good overall agreement with high-end ultrasound systems when assessing some, but not all, abdominal pathologies. Patients were seen in the emergency ward [18] or in internal medicine wards as in- or outpatients [14,15,16,17].

Four of the included studies assessed the use of hand-held ultrasound devices for diagnosing ascites [15,16,17,18]. One of these studies also investigated the best site for paracentesis, and found 100% agreement for determining the paracentesis location [17]. All studies concerning the presence of ascites showed good agreement between high-end ultrasound systems and hand-held devices. For example, when diagnosing ascites using the “Focused Assessment with Sonography for Trauma” (FAST) method, Coşkun et al. found a statistically significant correlation between hand-held devices and high-end systems [18].

One study aimed to assess the accuracy of hand-held ultrasound for diagnosing cholelithiasis in patients referred to abdominal ultrasound because of symptoms for gallbladder disease [14]. The article showed high sensitivity (93.8%) and specificity (100%) when comparing the results of the expert operators. The conclusion of the study was that hand-held devices can be reliably used by experts for diagnosing cholelithiasis.

Other diagnostic attributes of hand-held devices were in the detection of fatty liver disease and parenchymal liver damage, but significant differences between hand-held devices and high-end systems have been found when measuring the size of the liver, spleen and kidneys [16]. In this study the liver was found to be on average 1.9 cm smaller on hand-held devices, and thus hepatomegaly was found in only 44% of cases. Spleen and kidney measurements on the hand-held devices were on average 0.4 and 0.6 cm smaller, respectively. Barreiros et al. found hand-held ultrasound useful for assessing complications after interventions and there was a high detection rate of 97% for various abdominal focal lesions larger than 2 cm [17].

### 3.6. Urology (Two Articles)

Of the two studies concerning the use of hand-held ultrasound on patients referred to urology wards, both showed good overall agreement when compared to high-end ultrasound systems [19,20]. Lavi et al. found differences to be insignificant when assessing kidney length, renal pelvis length, renal cyst diameter, post-void residual and prostate volume in a small group of patients admitted to a urology ward [20]. Kameda et al. assessed the performance of hand-held ultrasound for identifying and grading the presence of hydronephrosis in 200 kidneys in 100 patients and found the agreement between hand-held and high-end systems to be excellent [19]. Barreiros et al., included in the abdominal section of this article, also found excellent agreement between the systems for diagnosing hydronephrosis [17].

### 3.7. Obstetrics and Gynaecology (Four Articles)

Four articles evaluated hand-held devices for obstetric ultrasound, two of which also included gynaecological patients. Patients were either seen for a routine check-up [22,24], seen in hospital because of an acute illness [21] or referred to ultrasound from another physician [23,24]. Of the 4 articles concerning obstetrics and gynaecology, all four articles found hand-held devices to generally be in strong agreement with high-end ultrasound systems.

For pregnant women in their first trimester there were no disagreements between hand-held devices and high-end systems when visualising the presence of an embryo, the gestational sac, the embryo heart beat or when comparing foetal measurements [21,23,24]. For women in their second and third trimester there were no disagreements when identifying and measuring any target structures [22,24]. Similarly, among gynaecological patients, hand-held devices were in good overall agreement with high-end systems for detecting leiomyoma, endometrial polyps, ovary follicles, ovary neoplasias, ascites, as well as for measuring target structures, apart from endometrial thickness, with measurements consistently larger on hand-held devices [23,24]. Bruns et al. observed a low correlation for diagnosing ectopic pregnancies, however with only a few cases, they provided no definitive conclusions [21].

### 3.8. Vascular (Three Articles)

All three studies with a vascular focus examined the abdominal aorta, and all included articles found hand-held ultrasound to be a reliable tool for determining the abdominal aortic diameter and for the early detection of abdominal aortic aneurysms. Two articles investigated the use of a hand-held device as a screening tool for detecting abdominal aortic aneurysms when considering the impact of cardiovascular risk factors or as a direct follow up after cardiac disease [25,27]. There were strong correlations between hand-held devices and high-end ultrasound systems in both articles. Bonnafy et al. [26] evaluated the ability of hand-held devices to assess aortic diameters in patients initially hospitalised for cardiovascular disease, concluding that the abdominal aorta can be accurately measured with a hand-held ultrasound device.

### 3.9. Bias and Applicability

The articles included have been inspected for risks of bias and applicability by the two authors (A.R. and J.F.C.) using QUADAS-2 [11] (Table 2). For a detailed presentation of our evaluation please see Supplementary Table S1.

## 4. Discussion

This systematic review shows hand-held ultrasound devices to be in overall agreement with high-end systems across several medical specialties when limited to distinct clinical questions. Several studies show good overall agreement for hand-held devices when detecting ascites, and they may prove to be a valuable bedside supplement to physical examination, or in emergency medicine, when performing Focused Assessment with Sonography for Trauma (FAST) [15,16,17,18]. Strong correlations were also found for obstetric and gynaecological patients [21,22,23,24] for examining the pleural cavities [12,13], detecting hydronephrosis [17,19], and screening for abdominal aortic aneurysms/measuring the aortic diameter [25,26,27]. Some applications were only considered in a single article each, but they showed good overall agreement between the devices for detecting pathologies such as cholelithiasis [14] and for determining the best site for paracentesis [17]. Hand-held devices were generally found to be inferior to high-end ultrasound systems when assessing superficial structures, i.e., in oncology, when estimating vascularity, ectopic pregnancies, and in the examinations of obese patients [17,21]. As for the smaller average sizes of liver, spleen and kidneys found in one study, it was explained this could be due to the different transducers used and their physical properties [16]. One article found larger values on hand-held ultrasound devices when measuring endometrial thickness, which is explained could be due to the fact that these measurements are smaller and more likely to be affected by an empty bladder and from using an abdominal probe [20].

Further potential clinical applications of hand-held ultrasound have been examined in a position paper for the European Federation of Societies for Ultrasound in Medicine and Biology (EFSUMB) [28], discussing the utility of hand-held ultrasound in abdominal, echocardiographic, lung and paediatric ultrasound, as well as for use in the training of medical students. This paper concludes that hand-held ultrasound is primarily to be used in point-of-care ultrasound with narrowly defined examination objectives.

While the aim of this systematic review has been to see how hand-held ultrasound performs in direct comparison with high-end systems, it could be relevant to consider how the use of hand-held ultrasound would influence the workflow in clinical settings. Some studies have found that by adding hand-held ultrasound to the routine of a clinical examination in patients admitted to medical wards, it either confirmed, changed or added important diagnoses in up to 1 of 3 patients [29,30]. This could potentially reduce the time before a diagnosis is made [31]. In a study of 1962 patients seen in different clinical settings by either general practitioners or specialists, hand-held ultrasound confirmed the initial clinical hypothesis in 66% of patients and could reduce the need for further testing [4]. A systematic review by Becker et al., on the use of hand-held and portable devices in low-and middle-income countries, found that hand-held ultrasound may have an impact on clinical management in up to 70% of cases, so that hand-held ultrasound can be used to triage, diagnose and treat a variety of patients when a high-end system is not available [32]. However, the quality of the evidence was low and larger clinical trials are needed.

There were big discrepancies in the level of experience among operators in the included studies. While some studies had experienced operators performing all ultrasound examinations, other studies compared the results of expert physicians using high-end ultrasound systems with those of non-expert nurses, family physicians, medical residents or medical students using hand-held ultrasound devices. The results of one group of study participants cannot necessarily be transferred to other groups and comparing the results of articles using operators with many different levels of experience might not give an accurate representation of the performance of hand-held ultrasound devices. The pre-study training periods also varied, adding to the heterogenicity of the included studies. Since this systematic review spans across several medical specialties with many different uses of hand-held devices, it is possible that the use of hand-held ultrasound devices should be a job for specialists in some scenarios, whilst in other instances could be outsourced to non-specialists. 

The definition of a hand-held ultrasound device is not straightforward. We found that whilst many studies did compare portable ultrasound devices with high-end machines, the portable devices in question were in fact the size of laptop computers. In this review, devices described as being able to fit a physician’s coat pocket were considered true hand-held devices. There is an increasing number of hand-held devices on the market, all vary significantly in their capabilities, so each model might have its own advantages [33]. In the studies included in this review, only two different devices were used: Fifteen using Vscan (GE Medical) and one using Acuson P10 (Siemens) [16]. Vscan is the most recent of these two hand-held devices, it is smaller and also provides colour Doppler imaging. Table 3 provides a short summary of the specifications of these devices. Since the release of Vscan and Acuson P10, several newer hand-held devices have entered the market offering new functions such as wireless transducers that connect to a tablet or a smartphone. However, studies on the comparative capabilities of such new devices did not appear in our search of the literature.

Some studies in our search used smaller, and sometimes portable systems for reference imaging and was not included in this review. The high-end ultrasound systems used for reference imaging in the included articles of this review were required to be large systems that generally offer excellent image quality with a high flexibility in function. 

This review was limited to the abdominal and pleural applications of hand-held ultrasound. In another systematic review by Galusko et al. they examined studies related to the echocardiographic use of hand-held ultrasound devices [36]. A total of 25 studies were included for analysis. The studies had to include sensitivities or specificities on the results of the hand-held device and were divided into three groups based on experience: Those carried out by experienced users, by users with little experience in ultrasound and by nurses. All groups achieved high diagnostic parameters for detecting cardiac pathology, and it was concluded that hand-held ultrasound can be used as a screening tool and offer better diagnostic capabilities than physical examination for cardiac pathology, but data was highly heterogenous with tests done in various settings and on different categories of patients. Not all of the included studies reported comparisons with standard transthoracic ultrasound. 

Two articles not included in this review showed good overall agreement for detecting B-lines in patients with heart failure, as well as for visualising the diaphragm [37,38]. Some articles concerning hand-held ultrasound in abdominal pathology were not limited to comparing hand-held ultrasound devices with a specific high-end system [29,39,40,41], but were compared with other diagnostic measures, such as physical examination, CT and MRI, as well as US and were not included in this paper.

The studies in this review varied greatly in a number of ways including different settings, operator experience, patient categories and different anatomical areas, with only a few articles covering each area. Our QUADAS-2 analysis suggests that there is no blinding, or description of blinding in eight of the included articles [16,17,18,20,21,23], making the articles prone to bias. Overall there was little risk of bias relating to patient selection, and although it was unclear in some studies whether there had been an appropriate time interval between the index tests and reference standards [13,15,16,17,26,27], all patients were included for analysis receiving the same reference standard. There were almost no concerns regarding applicability of the included studies for this review.

This review has focused on hand-held ultrasound devices compared to high-end ultrasound machines. In the future, hand-held devices may find new diagnostic territories when combined with other evolving medical and non-medical technologies. Cloud-based image analyses and storage in combination with a 5G internet connection could, in combination with light and affordable hand-held devices, make ultrasound examinations available in remote locations far from specialised hospital wards. Image interpretation could be performed in real-time by experienced ultrasonographers at a distance, allowing for immediate and optimal diagnoses even for patients far away from the expert physician. New robotics technology could allow for probe placement performed by remote sonographers, and even biopsy and paracentesis could be performed by a distant physician experienced in interventional ultrasound procedures [42].

Further, artificial intelligence (AI) has been proposed as a new technology to speed up and improve ultrasound image acquisition, and even help physicians with image interpretation. AI could make the image quality of hand-held devices more akin to that of high-end systems, and potentially aid the dissemination of ultrasound to more physicians, as image analysis could be aided by a non-human ultrasound expert [43].

## 5. Conclusions

In conclusion, the articles included in this review have found hand-held ultrasound devices to be in good overall agreement with high-end ultrasound systems when limited to distinct clinical questions such as in the detections of ascites and hydronephrosis, when screening for abdominal aortic aneurysms and for examining pleural cavities. Strong correlations were also found for obstetric and gynaecological patients. More possible applications were considered in the included articles, but these were only considered in a single article each. Due to the heterogeneity of the relatively few studies included, the evidence is inadequate which makes it difficult to draw definitive conclusions. More research is needed on the abdominal and pleural applications of hand-held devices with more standardised comparisons using only blinded reviewers.

## Figures and Tables

**Table 1:** Overview of included studies grouped by anatomical areas. (*) Hand-Held Device.

	Year	Author	Study Aim	Site of Interest	Patients	HHD(*)	High-End Ultrasound	Operator Experience	Results	Conclusion
**PLEURA**	2015	Dalen et al. [12]	To study the feasibility and reliability of focused hand-held ultrasound examinations of the pleural cavities and the inferior vena cava performed by nurses	Pleural cavities and inferior vena cava	62 heart failure patients	Vscan, GE Medical	Vivid 7, GE Medical	HHD by specialised nurses after dedicated training. High-end system by cardiologist	Sensitivity, specificity, positive and negative predictive values ≥92%, and correlations with reference were high with all measurements	Specialised nurses were, after a dedicated training protocol, able to obtain reliable recordings of both pleural cavities and the inferior vena cava by HHD and interpret the images in a reliable way
	2015	Graven et al. [13]	To study the feasibility and reliability of focused ultrasound to quantify pericardial and pleural effusion by an HHD performed by nurses	Pericardial and pleural cavities	59 patients early after cardiac surgery	Vscan, GE Medical	Vivid E9, GE Medical	Cardiac nurses with 3 months of training with an HHD. Reference imaging on a high-end system by 1 of 4 cardiologists	The correlations of the degrees of pericardial and pleural effusions in comparison with reference were *r* = 0.76 and 0.81, respectively	Cardiac nurses were able to obtain reliable measurements and quantification of both pericardial and pleural effusion bedside by HHD
**ABDOMEN**	2018	Del Medico et al. [14]	To investigate the accuracy of HHD in diagnosing cholelithiasis	Gallbladder	146 patients referred with symptoms of gallbladder diseases	Vscan, GE Medical	Alpha 6 Prosound, Hitachi or Esaote MyLab 70, XVG	Expert operators on both HHD and high-end systems. Non-experts on HHD	With experts using HHD, sensitivity and specificity were 93.75 and 100%, respectively. Sensitivity and specificity by non-experts were up to 93 and 88%	HHD showed a high diagnostic accuracy in diagnosing cholelithiasis when performed by expert operators
	2017	Andrea et al. [15]	To assess the efficacy of a brief teaching program using an HHD focusing on the bed side diagnosis of subclinical ascites	Abdominal free fluid	5 cirrhotic patients without ascites and 5 with subclinical ascites	Vscan, GE Medical	Aloka Alfa-10	HHD by five post graduate medical doctors. High-end system by expert sonographer and medical doctor	The students made no false positive diagnosis of ascites, and one false negative of subclinical ascites	The use of HHD for diagnosis of subclinical ascites in the context of a short, structured teaching program was efficient with no false positive results
	2015	Stock et al. [16]	To investigate the accuracy and time savings of HHD compared with high-end systems	Various abdominal pathologies	28 hospitalised patients on the ward at bedside	Acuson P10, Siemens	Sonoline Antares	Two internal medicine specialists, experienced in ultrasonography	82 of 113 pathological findings were detected with HHD. Measurements of liver, spleen and kidney differed significantly	The clinical utility of HHD is limited. Useful for distinct clinical questions such as detection of ascites and pleural effusion when used by experienced examiners
	2014	Barreiros et al. [17]	To assess image quality, indications and limitation of HHD compared with high-end systems	Abdominal focal lesions, ascites, etc.	231 patients requiring an US examination of the abdomen	Vscan, GE Medical	Logiq E9, GE Medical	Two experienced physicians	Image quality was considered sufficient in 97.4%. 97% of abdominal focal lesions and 94.7% with diffuse disease (i.e., hydronephrosis) were detected. 100% agreement on best site for puncture in patients with ascites	The investigated HHD displays a sufficient image quality, in some indications such as abdominal focal lesions >20 mm, ascites detection and hydronephrosis
	2011	Coşkun et al. [18]	To investigate the usability and the reliability of HHD in determining free fluid during the initial evaluation of trauma patients	Abdominal free fluid	216 trauma patients	Vscan, GE Medical	SSA660A/Nemio 10, Toshiba or Sonoline G4, Siemens	Emergency physicians with 4 hours training in Vscan and 4 hours simulation training. High-end systems by radiologists	Vscan sensitivity for determining free fluid was 88.9%, specificity 97.6%, negative predictive value 99.5% and positive predictive value 61.5%	Statistically significant correlation between the results of FAST performed by emergency physicians using HHD and the results by radiologists on high-end systems
**UROLOGY**	2018	Kameda et al. [19]	To assess HHD for evaluating dilatation of the renal collecting systems	Kidney	200 kidneys in 100 patients	Vscan, GE Medical	SSA680A/SSA780A/SSA790A/Aplio500, Toshiba	Eleven sonographers with at least 2 years’ experience	Excellent agreement between devices with sensitivity up to 91%	HHD useful for evaluating hydronephrosis when used by skilled sonographers
	2017	Lavi et al. [20]	To evaluate the utility of HHD and to assess quality of a urologist-performed study	Kidney, bladder and prostate	36 patients admitted to the urology ward for various reasons	Vscan, GE Medical	GE Volusion 730/Logic Q8, GE Medical	HHD by urologist. High-end system by sonographer	Differences in measurements were found to be insignificant with high interobserver agreement for evaluating hydronephrosis	HHD can be used by urologists to evaluate the upper and lower urinary tract with the exception of renal masses
**OBSTETRICS AND GYNAECOLOGY**	2015	Bruns et al. [21]	To determine the applicability of HHD as a complementary method for clinical evaluation during the first trimester of pregnancy	Embryo and intrauterine gestation	86 pregnant women in their first trimester attended in an emergency	Vscan, GE Medical	Voluson 730 Expert, GE	6 professionals classified as ultrasound-specialists in OB-GYN. Comparison between devices by the same physician	Best comparative results were for visualising the embryo heartbeat with a kappa coefficient of 0.84. Low correlation for detecting ectopic pregnancies	Potential for HHD to become a complementary and accessible diagnostic tool in obstetric patients during the first trimester. Not to use for ectopic pregnancies
	2014	Galjaard et al. [22]	To evaluate the application of HHD in a routine antenatal third-trimester scan compared with a high- end system	Foetal growth, well-being and position	50 unselected patients who came for a routine third-trimester US-scan	Vscan, GE Medical	Voluson 730 Expert, GE	HHD by experienced operator. High-end system by an experienced ultra-sonographer	Perfect agreement for foetal position, foetal bladder and visualising the stomach. Very good agreement for placental position. Good agreement for foetal growth measurements	HHD proved to be a reliable alternative to the high-end system for diagnostic evaluation in late pregnancy
	2013	Troyano Luque et al. [23]	To validate a new clinical OB-GYN application for HHD. Vscan was modified and tested for transvaginal use	Embryo, endometrium and ovaries etc.	80 patients referred for transvaginal- ultrasound: 25 obstetric and 55 gynaecological	Vscan, GE Medical	Voluson 730 Expert, GE	All examinations were carried out by the same specialist with 25 years of experience	The total detection rate of lesions with HHD was 98.75%. Measurements with HHD were 0.3–0.4 cm lower than those obtained with a high-end system	A novel transvaginal application of HHD demonstrates detection capabilities comparable to high-end systems
	2012	Sayasneh et al. [24]	To evaluate the performance and potential impact on patient management of HHD in comparison with a high-end system	Embryo, endometrium and ovaries, etc.	204 patients in 3 categories: Problems during early pregnancy, routine obstetric US and gynaecological pathologies	Vscan, GE Medical	Voluson E8 Expert, GE	Examiners were divided in 4 groups depending on their level of experience ranging from specialist medical staff to the junior ultrasound trainees	Good to very good agreement in obstetric ultrasound. Very good agreement for the evaluation of ovarian masses. Close agreement between measurements, except for endometrial thickness	Images obtained with HHD is in close agreement with those obtained using a high-end system
	2017	Esposito et al. [25]	To assess the impact of demographics and cardiovascular risk factors on abdominal aorta size by using HHD in an outpatient screening	Abdominal aorta	513 patients, referred for a cardiovascular assessment in a 6 months period were screened	Vscan, GE Medical	Vivid 7, GE Medical	Blinded expert ultrasound operators on both HHD and the high-end system	The correlation with reference for measuring the abdominal aortic diameter was excellent, *r* = 0.97	Excellent agreement between HHD and a high-end system, suggesting that HHD could be a reliable tool for the screening of abdominal aortic aneurysms
**VASCULAR**	2013	Bonnafy et al. [26]	To assess the agreement between abdominal aortic diameter measurements performed by novice operators using HHD and those made by experts using high-end systems	Abdominal aorta	56 patients, initially hospitalised for cardiovascular diseases other than aortic disease	Vscan, GE Medical	iE33, Phillips	2 experts using high-end systems. 1 expert using and at least one medical student using an HHD	The intraclass correlation coefficients were all >0.91 and mean differences between measurements were <1 mm. Differences between experts and novices were <4 mm in 92% of cases	For the purpose of screening for abdominal aortic aneurysms the aortic diameter can be accurately measured with an HHD by novices after a short period of training
	2012	Dijos et al. [27]	To evaluate the accuracy of HHD for identifying abdominal aortic aneurysms when compared with a high-end system	Abdominal aorta	52 patients in the first stage of the study comparing HHD with high-end	Vscan, GE Medical	iE33, Phillips	Experienced physician using a high-end system followed by a blinded expert physician using an HHD	The detection rate of abdominal aortic aneurysms for HHD was 100%. Measurements were obtained of the aortic diameter with a 97.5% accuracy	Screening for abdominal aortic aneurysms using an HHD by an expert is promising. Could be used as an extension to the routine physical examination

**Table 2:** Evaluation of risk of bias and applicability of studies included in the analysis. For details see Supplementary Table S1.

Study	Risk of Bias				Applicability Concerns		
	Patient Selection	Index Test	Reference Standard	Flow and Timing	Patient Selection	Index Test	Reference Standard
Dalen et al. [12]	Low	Low	Low	Low	Low	Low	Low
Graven et al. [13]	Low	Low	Low	Low	Low	Low	Low
Del Medico et al. [14]	Low	Low	Low	Low	Low	Low	Low
Andrea et al. [15]	High	Low	Low	Low	Low	Low	Low
Stock et al. [16]	High	Low	Unclear	Low	Low	Low	Low
Barreiros et al. [17]	Low	Low	Unclear	Low	High	Low	Low
Coşkun et al. [18]	Low	Low	Unclear	Low	Low	Low	Low
Kameda et al. [19]	Low	Low	Low	Low	Low	Low	Low
Lavi et al. [20]	Low	Low	Unclear	Low	Low	Low	Low
Bruns et al. [21]	Low	Low	High	Low	Low	Low	Low
Galjaard et al. [22]	Low	Low	Low	Low	Low	Low	Low
Troyano et al. [23]	Low	Low	High	Low	Low	Low	Low
Sayasneh et al. [24]	Low	Low	Low	Low	Low	Low	Low
Esposito et al. [25]	Low	Low	Low	Low	Low	Low	Low
Bonnafy et al. [26]	Low	Low	Low	Low	Low	Low	Low
Dijos et al. [27]	Low	Low	Low	Low	Low	Low	Low

**Table 3:** A summary of the specifications of Acuson P10 and Vscan [34,35].

Hand-Held Ultrasound Device	Release	Weight	Screen Size	Features	Transducer Type	Frequency Range	Price
Acuson P10 (Siemens)	2007	700 g	3.7-inch	2D-mode (fundamental and harmonic)	Phased array transducer	2–4 Mhz	Approx. 4000 USD
Vscan (GE)	2010	400 g	3.5-inch	Black/white imaging as well as colour coded overlay	Phased array transducer and Linear array transducer	Phased (1.7–3.8 Mhz) Linear (3.4–8 Mhz)	Approx. 4000 USD

## Supplementary Materials

The supplementary table is available online at https://www.mdpi.com/2075-4418/9/2/61/s1.
