# Supplementary material for: Hand-Held Ultrasound Devices Compared with High-End Ultrasound Systems: A Systematic Review
Source: Diagnostics (Basel). 2019 Jun 15;9(2):61. doi: 10.3390/diagnostics9020061 (PMC6628329; doi:10.3390/diagnostics9020061)
Supplement: Supplementary file 1 [file diagnostics-09-00061-s001.pdf]

SUPPLEMENTARY MATERIAL

QUADAS-2 SCORE CHART	Dalen et al. 2015 [17]	Graven et al. 2015 [18]	Medico et al. 2018 [19]	Andrea et al 2017 [20]	Stoc k et al. 2015 [21]	Barreiros et al. 2014 [22]	Coskun et al. 2011 [23]	Kameda et al. 2018 [24]	Lavi et al. 2017 [25]	Bruns et al. 2015 [26]	Galjaard et al. 2014 [27]	Troyano et al. 2013 [28]	Sayasneh et al. 2012 [29]	Esposito et al. 2017 [30]	Bonnafy et al. 2013 [31]	Dijos et al. 2011 [32]
**DOMAIN 1: PATIENT SELECTION**																
**A. Risk of Bias**																
Was a consecutive or random sample of patients enrolled ?	Y	Y	Y	N	U	Y	Y	Y	Y	Y	Y	Y	Y	Y	Y	Y
Was a case-control design avoided?	Y	Y	Y	N	Y	Y	Y	Y	Y	Y	Y	Y	Y	Y	Y	Y
Did the study avoid inappropriate exclusions?	Y	Y	Y	U	U	Y	Y	Y	U	Y	Y	Y	Y	Y	Y	Y
Could the selection of patients have introduced bias?	L	L	L	H	H	L	L	L	L	L	L	L	L	L	L	L
**B. Concerns regarding applicability**																
Is there concern that the included patients do not match the review question?	L	L	L	L	L	H	L	L	L	L	L	L	L	L	L	L
**DOMAIN 2: INDEX TEST**																
**A. Risk of Bias**																
Were the index test results interpreted without knowledge of the results of the reference standard?	Y	Y	Y	N	Y	Y	Y	Y	Y	Y	Y	Y	Y	Y	Y	Y
If a threshold was used, was it pre-specified?	N/A	Y	N/A	N/A	N/A	N/A	N/A	Y	U	N/A	N/A	N/A	N/A	N/A	Y	Y
Could the conduct or interpretation of the index test have introduced bias?	L	L	L	L	L	L	L	L	L	L	L	L	L	L	L	L
**B. Concerns regarding applicability**																
Is there concern that the index test, its conduct, or interpretation differ from the review question?	L	L	L	L	L	L	L	L	L	L	L	L	L	L	L	L

Table S1. A detailed presentation of our evaluation of risk of bias and applicability of the included studies. Abbreviations explained: Y = Yes; N = No; H = High; L = Low; U = Unclear; N/A = Not Applicable.

**QUADAS-2 SCORE CHART**	Dalen et al. 2015 [17]	Graven et al. 2015 [18]	Medico et al. 2018 [19]	Andrea et al 2017 [20]	Stoc k et al. 2015 [21]	Barreiros et al. 2014 [22]	Coskun et al. 2011 [23]	Kameda et al. 2018 [24]	Lavi et al. 2017 [25]	Bruns et al. 2015 [26]	Galjaard et al. 2014 [27]	Troyano et al. 2013 [28]	Sayasneh et al. 2012 [29]	Esposito et al. 2017 [30]	Bonnafy et al. 2013 [31]	Dijos et al. 2011 [32]
**DOMAIN 3: Reference Standard**																
**A. Risk of Bias**																
Is the reference standard likely to correctly classify the target condition?	Y	Y	Y	Y	Y	U	Y	Y	Y	Y	Y	Y	Y	Y	Y	Y
Were the reference standard results interpreted without knowledge of the results of the index test?	Y	Y	Y	Y	U	Y	U	Y	U	N	Y	N	Y	Y	Y	Y
Could the reference standard, its conduct, or its interpretation have introduced bias?	L	L	L	L	U	U	U	L	U	H	L	H	L	L	L	L
**B. Concerns regarding applicability**																
Is there concern that the target condition as defined by the reference standard does not match the review question?	L	L	L	L	L	L	L	L	L	L	L	L	L	L	L	L
**DOMAIN 4: FLOW AND TIMING**																
**A. Risk of Bias**																
Was there an appropriate interval between index test(s) and reference standard?	Y	U	Y	U	U	U	Y	Y	Y	Y	Y	Y	Y	Y	U	U
Did all patients receive a reference standard?	Y	Y	Y	Y	Y	Y	Y	Y	Y	Y	Y	Y	Y	Y	Y	Y
Did patients receive the same reference standard?	Y	Y	Y	Y	Y	Y	Y	Y	Y	Y	Y	Y	Y	Y	Y	Y
Were all patients included in the analysis?	Y	Y	Y	Y	Y	Y	Y	Y	Y	Y	Y	Y	Y	Y	Y	Y
Could the patient flow have introduced bias?	L	L	L	L	L	L	L	L	L	L	L	L	L	L	L	L

Table S1 (continued). A detailed presentation of our evaluation of risk of bias and applicability of the included studies. Abbreviations explained: Y = Yes; N = No; H = High; L = Low; U = Unclear; N/A = Not Applicable.
